# Addgene: Making Materials Sharing “Science As Usual”

**DOI:** 10.1371/journal.pbio.1001991

**Published:** 2014-11-11

**Authors:** Joanne Kamens

**Affiliations:** Addgene, Cambridge, Massachusetts, United States of America

## Abstract

Addgene (www.addgene.org) is a nonprofit organization that facilitates biomedical research and discovery by improving access to useful research materials and information. To fulfill this mission, Addgene works with hundreds of laboratories all over the world to collect high-quality published plasmids and data for the repository that can then be distributed to academic institutions and used to further research. Biological resource centers such as Addgene are an important part of the scientific infrastructure. They play a key role in helping scientists overcome logistical barriers to sharing, improving experimental reproducibility, and optimizing use of limited resources.

## Abstract 2

Addgene: Making Materials Sharing “Science As Usual” Addgene, the nonprofit plasmid repository, facilitates sharing of plasmids and data. Addgene's mission is to accelerate research and discovery by helping scientists collaborate.

## Scientific Community Needs

Journals and granting agencies require scientists to make their published reagents available to the scientific community, and many scientists are eager to disseminate the tools they have created for collaborations. However, logistical issues often impede the efficient sharing of materials. Fulfilling requests of popular, frequently requested items puts a financial and time strain on lab staff [1]. Shipping to some countries requires complicated customs forms and procedures. Legal agreements are desired by the universities to protect the senders and recipients, but standard technology transfer procedures significantly slow down the process of providing materials [2]. Finally, the normal turnover of lab personnel as students and postdocs move to new positions makes it difficult for materials to be found in the freezer and for associated data to be retrieved from lab notebooks. Addgene and a host of other biological resource centers work to help scientists overcome these barriers to materials sharing (Table 1) [3].

**Table 1:** Examples of biological resource centers.

Name	Website
American Type Culture Collection (ATCC)	ATCC.org
BACPAC Bacmid Repository	bacpac.chori.org
Bloomington Drosophila Stock Center	flystocks.bio.indiana.edu
Coli Genetic Stock Center	cgsc.biology.yale.edu/index.php
Developmental Studies Hybridoma Bank	dshb.biology.uiowa.edu
DNASU Plasmid Repository and Core	dnasu.org/DNASU
Fungal Genetics Stock Center	www.fgsc.net
PlasmID DNA Resource Core	plasmid.med.harvard.edu/PLASMID
Toolbox Plasmid Repository/BIOSS	www.bioss.uni-freiburg.de/toolbox/
Zebrafish International Resource Center	http://zebrafish.org/zirc

The scientific community agrees that independent confirmation of results is an important part of the scientific endeavor. There is increasing evidence that the publication and peer-review systems that should ensure reproducibility of data are not operating as they should [4]–[5]. Poor explanation of experimental methods, including insufficient description of reagents in publications, is a potential cause of data irreproducibility [6]–[7]. Standardization of well-characterized and properly identified reagents is one obvious way to improve reproducibility.

As science and data have gotten “big” in the last decade, laboratories have been generating increasingly large numbers of valuable reagents. Graduate students, technicians, postdocs, and principal investigators are designing, making, and testing plasmids for their studies. Those reagents are often used for a study or two and then stored in the freezer for posterity (or sometimes lost in the ice at the bottom of a freezer and never seen again). This is a huge waste at a time when laboratories are struggling with reduced funds and limited resources. Availability of high-quality, validated reagents at accessible prices enables laboratories with less funding to consider projects that might otherwise be inaccessible. Sharing of already generated reagents also accelerates the scientific process, enabling more rapid validation and extension of scientific findings.

## Benefits to Depositors

**Figure 1:**
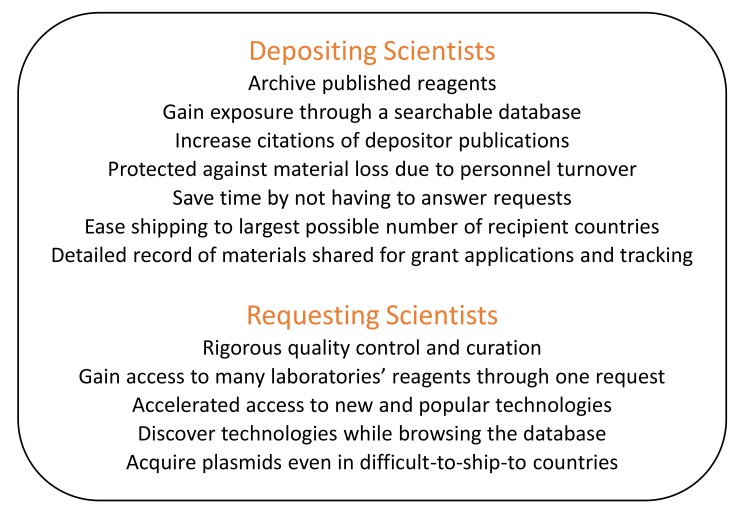
Benefits of sharing materials via a biological resource center.

Addgene works with over 2,000 depositing laboratories at 500 research institutions to make reagent sharing as easy as possible and with services that greatly benefit depositors (Figure 1). Addgene solicits plasmids by making lab visits around the world and by requesting deposits via email of plasmids from key publications. As awareness of Addgene has spread, an increasing number of scientists initiate deposits themselves by contacting Addgene to deposit their useful and published materials. Addgene employs approximately 20 PhD molecular biologists, quality control scientists, and lab technicians who work with depositors to receive samples, archive samples for tracking though our laboratory inventory management software, perform quality control, and then make as much data as possible available online.

Addgene also employs legal and administrative staff who work with technology transfer offices (TTOs) in the depositing institutions to arrange completion of a material transfer agreement (MTA) for each plasmid exchange. An MTA is a legal contract governing the sharing of research materials between two organizations. The MTA defines the rights of both the provider and the recipient with respect to the materials. The MTA process is largely handled by the Addgene online electronic MTA system, which was designed to make MTA completion as simple and rapid as possible. Addgene has developed working relationships with TTOs in most research institutions in the world, and TTO familiarity with the electronic MTA system greatly enhances the efficiency of technology transfer for plasmid sharing. Both depositing scientists and TTOs have special accounts on the Addgene website to view details of deposited and distributed materials. These data are useful for demonstrating the utility of research to the wider community for grant applications and publications.

Another advantage for depositing scientists is that the use of biological resource centers to share materials has been shown to result in an increase in citations [8]. Depositing scientists find it convenient to initiate a deposit before publication so that a unique Addgene accession number can be included in the materials and methods section of the paper. Data can be embargoed from the public database until the publication is released. Many scientists also deposit after publication or archive and share potentially useful plasmids that are unpublished. Each lab has its own page on the Addgene website that helps organize its materials by plasmid and by publication. This unique page makes it easy to refer requestors to their lab's plasmid collection. For more information on the deposit process at Addgene, please see the Frequently Asked Questions (FAQ) page at http://www.addgene.org/faq.

## Benefits to the Community

**Figure 2:**
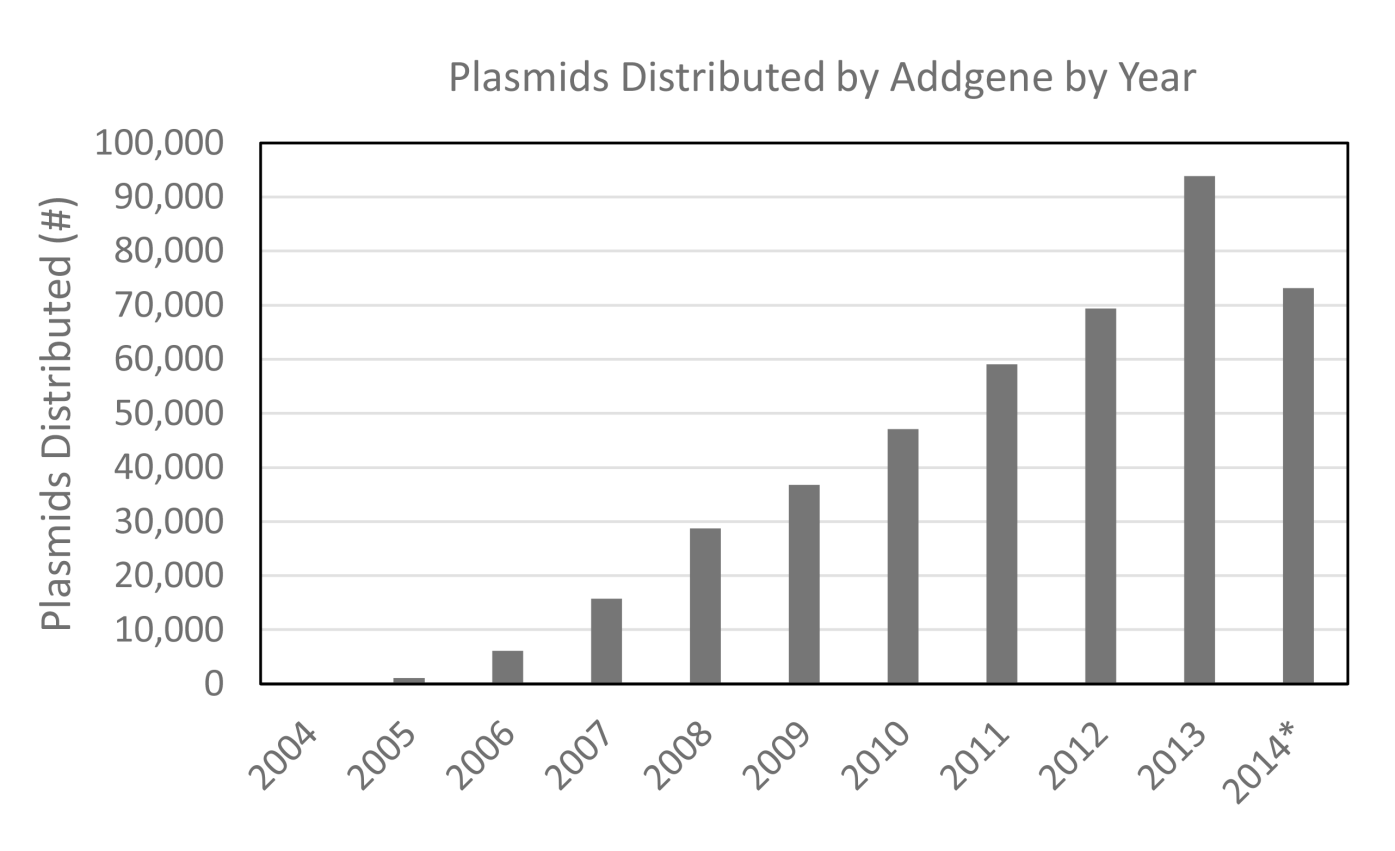
Distribution of plasmids from the Addgene library. The total number of plasmids and kits distributed by Addgene each year. For this analysis, multiplasmid kits were counted as “1” plasmid distribution. *2014 total reflects the number of distributed plasmids as of August 20, 2014.

As Addgene approaches the end of its tenth anniversary year of service, the pace of deposits is increasing, and distribution is robust. The repository now contains over 40,000 unique plasmids and distributes over 2,000 plasmids each week (Figure 2). The collection is always growing. In this way it remains pertinent and increases in value to the community. The steady increase in distribution has enabled Addgene to add staff to support the further growth of the library, maintain standards of quality control, and respond to the service needs of the community trying to access the plasmids and data. Members of the community work with Addgene to create educational resources on plasmid technologies. For examples, see the guide to optogenetics reagents (www.addgene.org/optogenetics) or the Addgene guide to choosing a plasmid backbone (https://www.addgene.org/empty_backbones). The participation of the entire community in the curation and presentation of the library enables Addgene to provide useful information for selection of reagents as well as educational resources for scientists new to molecular biology techniques.

Sequence data is an important part of the plasmid records. Addgene-generated sequence data and depositor-provided sequence and validation data are made available online. The Addgene quality control process includes sequence validation before distribution to ensure that key features (insert junctions, mutations, etc.) in the plasmid record are accurate. Both requesting and depositing scientists routinely contact Addgene with corrections and additions to the plasmid records. For example, a scientist might perform more extensive sequencing of a requested plasmid to plan a subcloning experiment and discover a discrepancy with the expected sequence. A depositor might generate new data that would be useful to associate with the plasmid record for future users. In these cases, new or updated information is confirmed by Addgene and reviewed with depositing scientists before being shared via the Addgene online database.

Addgene continues to work with partners to integrate its plasmid information into other databases. For example, in the PubMed database, an Addgene number in the online paper is a “hotlink” to the Addgene plasmid page, thus providing additional detail on the reagents used and adding value to the publication. Links to Addgene resources can also be found on a number of other resource sites, including NCBI LinkOut, Eagle-i (www.eagle-i.net), Neuroscience Information Framework (www.neuinfo.org), LabGuru (www.labguru.com), Nuclear Receptor Signaling Atlas (www.nursa.org), Snapgene (www.snapgene.com), Zebrafish Model Organism Database (http://zfin.org), and GeneCards (www.genecards.org).

**Figure 3:**
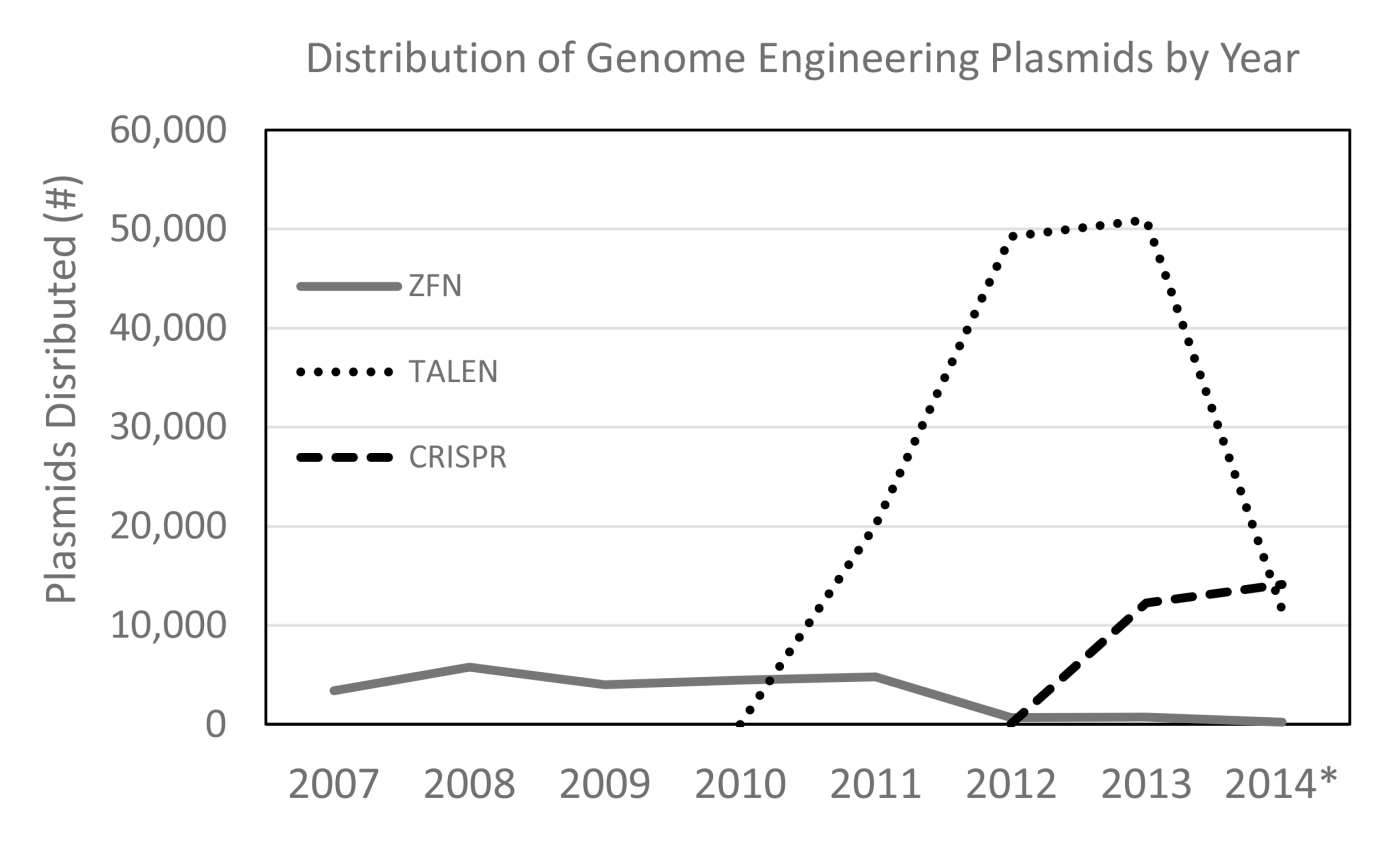
Dissemination of genome engineering technologies through plasmid sharing. Number of plasmids distributed for implementing zinc finger (ZFN)-, transcription activator-like effector nuclease (TALEN)-, or CRISPR-mediated genome engineering technologies. Note that various ZFN and TALEN assembly technologies require kits of plasmids ranging in size from 12 to as many as 834 plasmids (TALEN kit). CRISPR technology implementation requires a few individual plasmids. These data represent all plasmids distributed. *2014 totals reflect the number of distributed plasmids as of August 20, 2014.

One recent and telling example of the impact a repository can have is the Addgene distribution statistics for plasmids in the field of genome engineering, especially in the blossoming area of clustered regularly interspaced short palindromic repeats (CRISPR)/Cas9-mediated engineering. This technology can be used to modify endogenous DNA sequences in the genome of any organism. In January 2013, the first papers describing the use of CRISPR/Cas9 for genome engineering were published [9]–[11]. Many of the early leaders in this field were already Addgene depositing scientists. As noted in a recent review, “the rapid adoption of the Cas9 technology was also greatly accelerated through a combination of open-source distributors such as Addgene, as well as a number of online user forums” [12]. In another recent publication, a table listing of 43 different Cas9 variants included a column with the Addgene accession number for each of the variants—even those not yet published [13]. As of June 2014, Addgene maintains the most diverse collection of CRISPR/Cas9 reagents available to academic researchers, including 400 plasmids from 44 different labs. In just 18 months, Addgene has distributed over 23,000 CRISPR/Cas9-associated plasmids (Figure 3). The availability of these resources has enabled scientists all over the world to rapidly contribute to this fast-moving field.

## Conclusions

Addgene was established as an independent repository, with no reliance on a single university or funding agency, and has been self-sustaining since 2007. As such, it serves as one successful example of a repository that has resisted the financial crisis that research infrastructure organizations are currently facing [14]–[15]. The scale of the library is one of the factors in its success. By enabling the sharing of a large number of useful materials at a low price, Addgene has been able to continue to serve the community without relying on grant support. This could serve as a model for smaller repositories, which may wish to combine into larger repositories for efficiency and sustainability. The scientific community benefits in many ways from robust sharing of materials via centralized repositories. Addgene strives to make it so easy that sharing can be just “science as usual.”
